# Microbial Enhance of Chitosan Production by *Rhizopus arrhizus* Using Agroindustrial Substrates

**DOI:** 10.3390/molecules17054904

**Published:** 2012-04-27

**Authors:** Antonio Cardoso, Clarissa Izabel M. Lins, Ednaldo Ramos dos Santos, Marta C. Freitas Silva, Galba M. Campos-Takaki

**Affiliations:** 1 Northeastern Network of Biotechnology (RENORBIO), Federal Rural University of Pernambuco, 50050-900 Recife, PE, Brazil; Email: antoniocardoso2000@yahoo.com.br; 2 Nucleus of Research in Environmental Sciences (NPCIAMB), Catholic University of Pernambuco, Boa Vista 50050-590 Recife, PE, Brazil; Email: rissabel@gmail.com (C.I.M.L.); santosmestrado@gmail.com (E.R.S.); martacfs@yahoo.com.br (M.C.F.S.); 3 Post-graduation Program of Chemical Engineering, State Campinas University, Campinas 13083-852, São Paulo, Brazil; 4 Post-graduation Program of Biological Sciences, Federal University of Pernambuco, Recife 50670-420 Pernambuco, Brazil

**Keywords:** *Rhizopusarrhizus*, chitosan, biopolymer, agroindustrial substrates

## Abstract

This study investigated the potential of *Rhizopus arrhizus* UCP 402 for producing chitosan using corn steep liquor and honey as agroindustrial nitrogen and carbon sources. A complete factorial design was used to assess the improved biomass and chitosan production. The results were evaluated using Pareto charts (Statistica 7.0 software). The chitosan obtained was characterized by X-ray diffraction. The cristallinity index (I_C_), and infrared spectroscopy (FTIR) were used to evaluate the degree of deacetylation (DD %). The morphological aspects of the *R. arrhizus* were evaluated by measuring the diameter of the colonies by light microscopy. The results obtained showed higher biomass and chitosan yields (20.61 g/L and 29.3 mg/g), respectively, in the selected assays. The characterization of the macromolecular arrangement of chitosan showed a crystallinity index compatible with the literature, and the infrared peaks confirmed a degree of 86%. The experimental data obtained suggest that adding honey to corn steep liquor is a promising way to improve microbiological chitosan production.

## 1. Introduction

The genus *Rhizopus* is formed by cosmopolitan, filamentous fungi, found in soil or rotting fruit, vegetables, animal feces and food. Their morphological aspects such as the length of rhizoids and sporangiophores, the sporangia diameters, the columella shape, size and texture of sporangiospores help to differentiate the various species of this genus [1,2]. Like other members of the Zygomycetes class the filamentous fungus *Rhizopus arrhizus* presents chitin and chitosan polymers in the structure of its cell wall and are a group that is of high interest for biotechnological applications [3]. Chitosan is a polymer consisting of monomeric β-(1-4)-D-glucosamine units that can be modified by several stages of deacetylation, giving rise to diverse derivatives [4]. Chitin and chitosan have versatile properties that allow the use of these polymers in different research fields such as those of the medical, pharmaceutical and environmental areas [3] and this has economic advantages. Recent studies showed that several fungal species have been used as alternative sources for chitin and chitosan production [5,6]. The use of fungi to obtain chitin and chitosan has shown great advantages such as the extraction of co-occurring biopolymers, regardless of seasonal factors, and its being a simple and economical process that results in reductions of the time and cost required for extraction. 

Microbiological studies are dependent on the ability to grow microorganisms and maintain them in a laboratory. The culture medium and environmental conditions are important factors for fermentation processes. Thus, the necessary conditions for microbiological growth include the availability of carbon and nitrogen sources of enzymes and inorganic substances such as minerals and water. In processes for obtaining fungal biomass through laboratory fermentation, traditional techniques are usually employed in which the substrates, from which carbon and nitrogen are obtained, essential among other factors for the culture of the microorganism, are of synthetic origin. These substrates induce the production of the biopolymers chitin and chitosan and this reduces production costs. Nowadays studies have been published on applying different biotechnological fungi to produce chitin and chitosan for biomass, especially the species belonging to the Zygomycetes class [7,8,9,10,11,12,13].

This study set out to investigate the production of biomass and chitosan by the filamentous fungus *Rhizopus arrhizus* UCP 402 using a 2^2^ factorial design, employing corn steep liquor and honey as variable factors with the production of chitosan as a variable response. The average effect on *R. arrhizus* morphology was evaluated by light microscopy and the diameter of radial growth.

## 2. Results and Discussion

### 2.1. Influence of the Culture Media on *R. arrhizus* Growth

**Figure 1:**
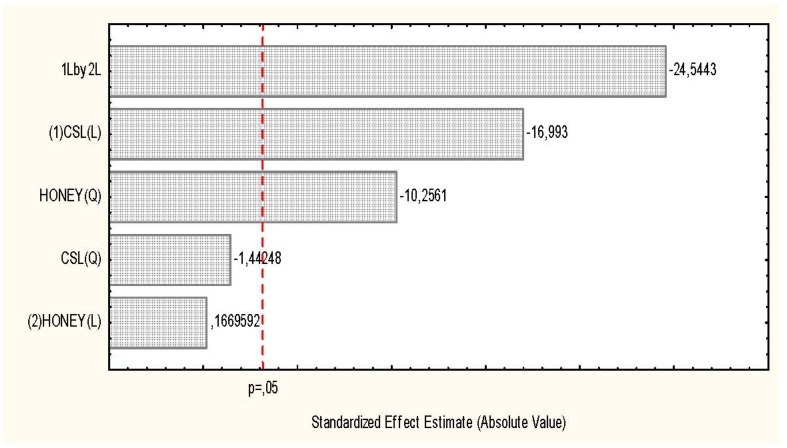
Pareto chart showing the effect of the culture medium on biomass production by *Rhizopus arrhizus*.

**Figure 2:**
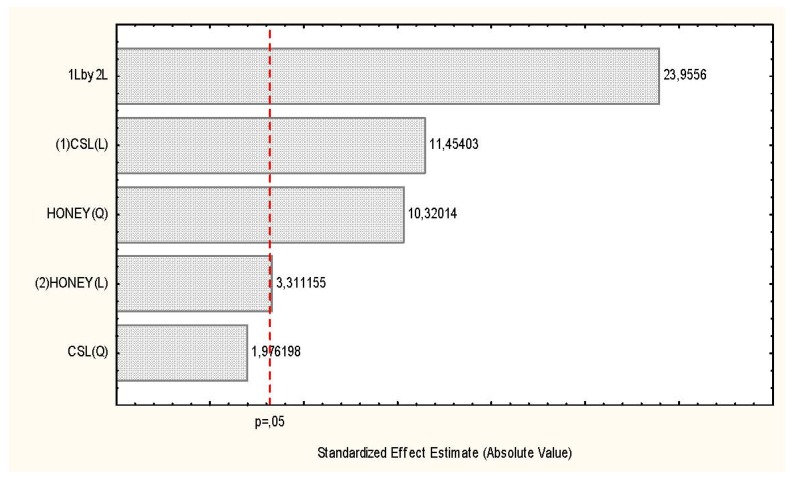
Pareto chart showing the effects of the carbon and nitrogen sources on chitosan production by *R. arrhizus*.

Table 1 and Figure 1 and Figure 2 present the results obtained for biomass, chitosan yield and the influence of carbon and nitrogen sources for *R. arrhizus* growth, and indicated that condition 2 gave the highest yield of mycelial mass produced (20.61 g/L) in culture media consisting of corn steep liquor (8%) and honey (6%) during 96 h of fermentation. It was observed that by the end of this period, consumption of the total reducing sugars present in the culture media used in this experiment was about 80%. At the same time, there was an increase in the pH range from 5.6 to 6.1. For chitosan production, the selected condition showed 29.3 mg/g of biomass (Table 1), in culture media comprising corn steep liquor (6%) and honey (13.24%). The results obtained in this study are corroborated in the literature [14,15], which describes an increase of mycelial mass by *Lyophylum decastes* using soybean seed as an alternative carbon and nitrogen source for producing extracellular polysaccharides, which gave a biomass yield of 5.6 g/L, a value that is lower than the yield for the best conditions selected in this study. 

**Table 1:** 2^2^ factorial design applied to biomass and chitosan production by *Rhizopus arrhizus* using corn steep liquor and honey as agroindustrial substrates.

Assays	Corn Steep liquor % (v/v)	Honey % (v/v)	Dried biomass (g/L)	Chitosan (mg/g)
1 2 3 4 5 6 7 8 9 10 11 12	4.00 8.00 4.00 8.00 3.17 8.83 6.00 6.00 6.00 6.00 6.00 6.00	6.00 6.00 12.00 12.00 9.00 9.00 4.76 13.24 9.00 9.00 9.00 9.00	14.83 20.61 14.21 8.26 12.75 13.80 11.51 11.71 14.91 14.48 14.71 14.36	24.00 7.40 19.80 23.90 20.00 21.20 17.60 29.30 18.10 18.30 18.90 17.96

The ability of *Cunninghamella bertholletiae* to produce chitosan was tested [5] using sugar cane juice and molasses as an alternative medium. The authors reported 10.5 g/L yields of biomass and 128 mg/g of chitosan. Stamford *et al*. [7] reported 24.3 g/L of mycelial mass of *Cunninghamella elegans* grown in yam bean (tubers) without additional carbon and nitrogen sources, this result was 15% higher than that found in this study. The nitrogen source influenced the increase in biomass and chitosan production by *Aspergillus niger* grown in soybean meal and canola oil [10], and the authors obtained a biomass and chitosan yields of 11.0 g/L and 120.5 mg/g, respectively. However, studies [6] testing the ability of *Absidia corymbifera* grown in submerged fermentation using corn steep liquor (6%) as the only source of carbon and nitrogen, and heavy metals (Cu^+^ and Zn^+^) obtained yields of 6.97 g/L of biomass and 67.29 mg/g of chitosan. This yield of chitosan is higher than that obtained in this experiment due to the influence of the heavy metals on the enzymatic action of chitin deacetylase, according to the authors.

### 2.2. Morphological Aspects of *R. arrhizus*

#### 2.2.1. Radial Growth of *R. arrhizus*

**Figure 3:**
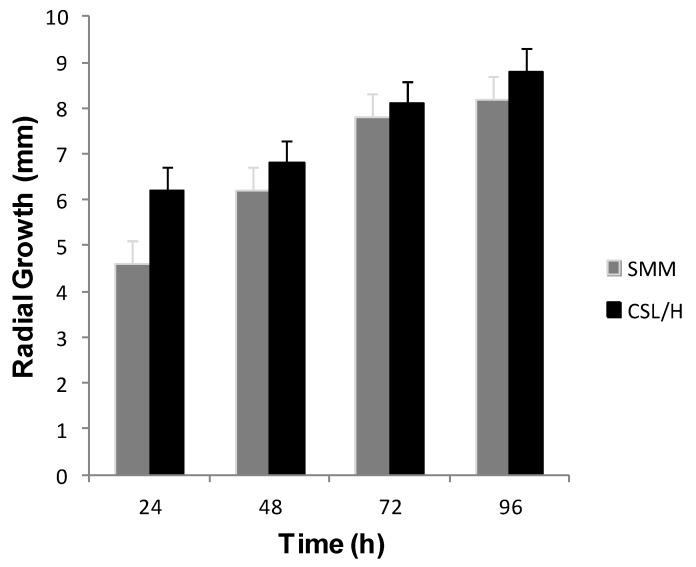
Radial growth of *Rhizopus arrhizus* in Synthetic Medium for Mucorales (SMM) and Corn Steep Liquor/Honey medium (CSLH).

Figure 3 presents the radial growth of *R. arrhizus* in Synthetic culture Medium for Mucorales (SMM) used as control, and the low-cost alternative media, corn step liquor and honey (CSLH). Mycelial growth of 1.6 mm was observed after the first 24 h of fermentation and an increase of about of 0.5 mm to the end of fermentation period in Petri dishes containing the CSLH medium, values 25% and 6% greater than the increases observed in samples control containing SMM media over the same period. These results suggest that the increase in mycelial growth of *R. arrhizus* is related to the availability of carbon and nitrogen sources present in the alternative medium.

#### 2.2.2. Morphological Characterization of *Rhizopus arrhizus*

**Figure 4:**
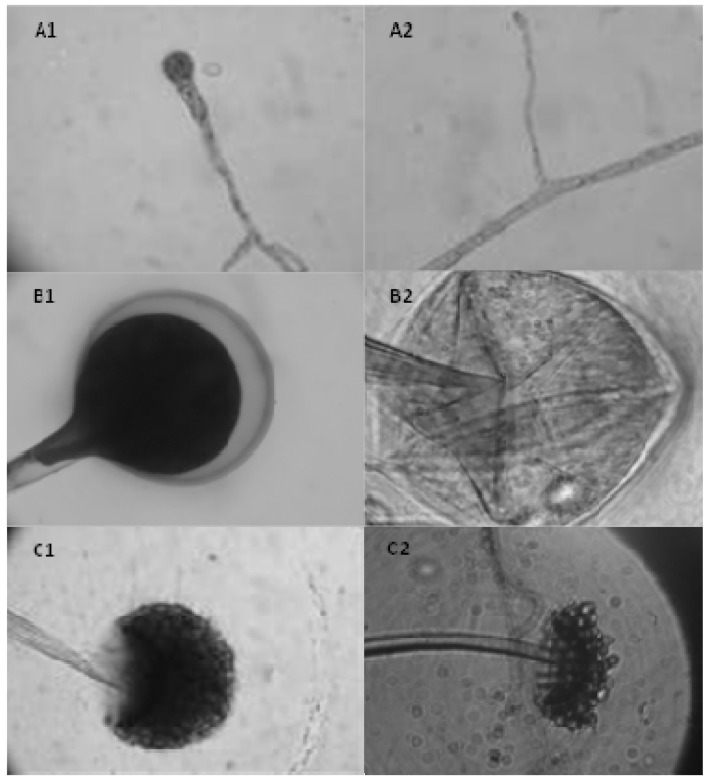
Light microscopy of *Rhizopus arrhizus* grown in Synthetic Medium for Mucorales-SMM (**A_1_**, **B_1_** and **C_1_**) and Corn Steep Liquor/Honey-CSLH (**A_2_**, **B_2_** and **C_2_**). (**A_1_**) Rhizoids and hyphae after 24 h of growth in SMM medium (100×); (**A_2_**) Hyaline hyphae after 24 h in CSL/H medium (100×); (**B_1_**) Sporangium covered with hyaline membrane, after 48 h of growth in SMM medium (100×); (**B_2_**) Mature sporangium released all sporangiospores, after 48 h of growth in CSL/H medium (100×); (**C_1_**) Sporangium with sporangiospores at 96 h in SMM medium (100×); (**C_2_**) Mature sporangium released sporangiospores at 96 h of growth in CSLH medium (100×).

The morphology of *R. arrhizus* grown in SMM medium are presented in Figure 4 (A_1_, B_1_ and C_1_) and for CSLH medium in Figure 4 (A_2_, B_2_ and C_2_), respectively. The morphological characteristics observed to SMM medium are corroborated by the literature [16,17,18]. On the other hand, different behavior was observed in *R. arrhizus* grown in corn steep liquor and honey medium (CSLH). Figure 4A_2_ shows the presence of hyaline hyphae and spores at 24 h, in CSLH medium. Figure 4B_2_ shows young sporangia containing hyaline membranes. However, in Figure 4C_2_ it is observed that mature sporangium and sporangiospores are released after 96 h of cultivation time, considering the reduction of nutrients available in the culture medium.

### 2.3. Characterization of Chitosan Extracted from the *Rhizopus arrhizus* Mycelium

**Figure 5:**
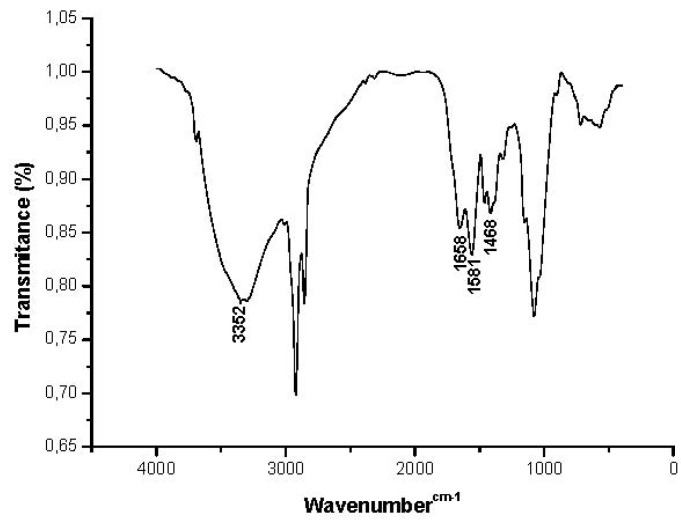
Infrared spectra of chitosan produced by *R. arrhizus* in alternative culture medium.

The characterization of isolated polymers is presented in Table 2 and Figure 5. This was carried out by X-ray diffraction and infrared spectra absorption. The results of chitosan cristallinity indices (65.97%) produced by *R. arrhizus* grown in the culture medium used, and with the selected conditions containing corn steep liquor and honey (CSLH) are presented in Table 2. The degree of polymer ordering was observed by X-ray diffraction analysis. This ordering can be influenced by several factors, mainly the isolation and lyophilization process [19,20].

**Table 2:** Characterization of chitosan samples produced by *Rhizopus arrhizus* in alternative culture medium.

Biopolymers	Infrared spectroscopy (FTIR)	Crystallinity index (%I_CR_)	Degree of Deacetylation (DD %)
Chitosan (Pattern) Chitosan (CSLHB) *	1,683, 1,596 and 1,455 cm^−1^ 1,658, 1,581 and 1,468 cm^−1^	64.00 65.97	83.00 86.00

* CSLH–Corn Steep Liquor and Honey.

The results calculated by the cristallinity index (I_CR_) are compatible with the literature [18]. The infrared spectra of chitosan extracted from the fungal biomass obtained from *R. arrhizus* grown in culture medium containing corn steep liquor (6%) and honey (13.24%), in the range of 86%, was higher than that observed in the sample of chitosan in the standard sample of chitosan, used as a reference for this study, and are consistent with those reported by the literature [8,9,18,19,20,21]. Peaks were observed at the corresponding chitosan amine bands, around 1,658, 1,581 and 1,468 cm^−1^ (Figure 5).

## 3. Experimental

### 3.1. Microorganism

*Rhizopus arrhizus* UCP 0402/WFCC 0402 was isolated from mangrove sediment, identified and deposited in the Culture Collection UCP (Universidade Católica de Pernambuco) of the Nucleus of Research in Environmental Sciences, Catholic University of Pernambuco, Brazil. The Culture Collection is registered in the World Federation Culture for Collection (WFCC), was maintained in PDA medium (Potato-Dextrose-Agar) at 5 °C. The culture was transferred to a new medium every four months.

### 3.2. Cultivation Conditions and Biomass Production

*R. arrhizus* grown in Petri dishes (9 cm/diameter) containing YMA culture medium (Yeast-Malt-Agar), consisting of agar (20 g), malt extract (3 g), peptone (5 g), glucose (10 g), distilled water (1,000 mL) and pH adjusted 5.8 was incubated at 28 °C for 5 days until sporulation. Spores were transferred to Erlenmeyer flasks (250 mL) containing distilled water to form a suspension of 10^4^ spores/mL. Aliquots (1 mL) were transferred to Petri dishes containing PDA medium for 24 h. Discs (40 × 0.6 mm/diameter) were transferred to Erlenmeyer flasks (1,000 mL) containing 400 mL of culture medium consisting of corn steep liquor and honey (CSLH), as per the independent variables and the factorial design (Table 1 and Table 3). The flasks were placed in an orbital shaker (150 rpm/28 °C/96 h). At the end of this period, the flasks were removed so as to estimate the production of biomass, to determine the pH and the consumption of glucose. The mycelial mass was removed by vacuum filtration, washed with distilled water, lyophilized and kept in a desiccator to gravimetrically determined constant weight. The biomass was stored for use in future stages of the experiments. The validation of the biomass and chitosan produced by *R. arrhizus* was undertaken on Pareto charts using the Statistica software (Figure 1 and Figure 2).

### 3.3. Determination of the Total and Reducing Sugars

The total and reducing sugars were estimated using metabolic cell-free liquid by the colorimetric method (DNS) described by Miller (1959) [22].

### 3.4. Determination Total Protein

The total proteins present in the metabolic cell-free liquid were estimated by the Bradford method [23].

### 3.5. Determination of pH

The values of pH were determined by means of a potentiometer.

### 3.6. Morphological Characterization of *R. arrhizus*

The morphological characterization of *R. arrhizus* was performed by slide culture (micro culture) and microscopic observation of mycelial growth every 24 h (Figure 3 and Figure 4). The synthetic culture medium for Mucorales (SMM) described by Hesseltine and Anderson [24], was used as a reference. The radial growth of the colonies was measured after every 24 h in a bacteriological incubator at 28 °C. These experiments were performed in triplicate and were observed using light microscopy.

### 3.7. Chitosan Extraction

Chitosan was extracted as described by the Hu *et al*. method [19], which consists of deproteinizing fresh biomass by adding sodium hydroxide solution (1 M/30 mL w.v/15 min/121 °C). The alkali insoluble fraction (AIF) was separated by centrifugation (4,000 rpm/15 min) and vacuum filtration. Dry biomass was obtained in alternating stages with saline solution (0.85%) and cold distilled water to pH 7.0. The residue obtained after this step was subjected to treatment with acetic acid (2%/30 mL w.v/15 min/100 °C), centrifuged (4,000 rpm) and filtered. The supernatant was alkalized to pH 9.0, stored in a refrigerator for 24 h and centrifuged (4,000 rpm/15 min) until the chitosan precipitated. The chitosan was washed with cold distilled water and saline solution to pH 7.0. Samples were placed in Petri dishes for drying (30 °C/24–48 h).

### 3.8. Chitosan Characterization

#### 3.8.1. Infrared Spectroscopy (FTIR)

The chitosan samples were previously dried overnight to 60 °C under reduced pressure and homogenized with 100 mg of potassium bromide (KBr). Discs prepared with potassium bromide were placed to dry for 24 h at 110 °C under reduced pressure. Infrared ray spectroscopy was performed using a Fourier Transform (FTIR) BRUKER Mod. IFS spectrophotometer. A blank potassium bromide disc was used as a reference. The maximum absorption bands intensity were measured by the baseline method.

#### 3.8.2. Degree of Deacetylation (DD %)

The degree of polymer deacetylation was determined as per the Roberts method [25], using the relationship between the absorbance (A1655/A3450), as shown in the equation:

DD % = (A1655/A3450). 100/1.33

#### 3.8.3. X-ray Diffraction

The chitosan X-ray diffractograms were obtained using a Siemens D5000 X-ray Diffractometer, CuKα radiation and α = 1.542Å, in a scanning range between 2° and 60° at a rate of 0.02° min^−1^. The interplanar distance was determined by the width of the half peak height of greatest intensity (I_CR_) The cristallinity index (I_CR_) was determined using the following equation: % = I_CR_(I_C_ − I_A_/I_C_) × 100. Where I_C_ and I_A_ are the intensities of the signals of the crystalline regions (20 = 20°) and amorphous regions (20 = 12°).

### 3.9. Factorial Central Composite Rotational Design (FCCRD)

In these experiments a factorial design with a Central Composite Rotational Design (CCRD) was used for selection the best condition for mycelial production and chitosan income in accordance with the variables established (Table 3).

**Table 3:** Independent variables used in 2^2^ full factorial design.

Independent Variables	Levels				
	+1.41	+1	0	−1	−1.41
Corn Steep Liquor (CSL) % (v/v)	8.83	8	6	4	3.17
Honey (H) % (v/v)	13.24	12	9	6	4.76

## 4. Conclusions

The results of this study show the potential of *R. arrhizus* of converting industrial waste (corn steep liquor) and honey to grow and produce chitosan. The alternative and low-cost medium used in this experiment can be used to obtain bioproducts, thus reducing operating costs. Similarly, *R. arrhizus* can be indicated as a quality source for obtaining chitosan, given the high levels of cristallinity (I_C_) and the degree of deacetylation (DD %), and also due to their metabolic efficiency and the ease with which this organism can be handled in laboratory assays.
